# MicroRNA-32-5p inhibits epithelial-mesenchymal transition and metastasis in lung adenocarcinoma by targeting SMAD family 3

**DOI:** 10.7150/jca.48387

**Published:** 2021-02-22

**Authors:** Jin-Xing Zhang, Wei Yang, Jun-Zheng Wu, Chun Zhou, Sheng Liu, Hai-Bin Shi, Wei-Zhong Zhou

**Affiliations:** Department of Interventional Radiology, The First Affiliated Hospital of Nanjing Medical University; Gulou, Nanjing 210029, P.R. China.

**Keywords:** non-small cell lung cancer, microRNA-32-5p, SMAD3, migration, invasion

## Abstract

Non-small cell lung cancer (NSCLC) is the leading cause of cancer-associated death worldwide. MicroRNA (miRNA)-32-5p is as an important cancer-associated miRNA in different types cancer. To date, the role of miR-32-5p in the migration and invasion of NSCLC remains unknown. In the present study, a Transwell assay was performed to investigate the role of miR-32-5p in lung adenocarcinoma. miR-32-5p expression level was determined via reverse transcription-quantitative PCR in 24 pairs of NSCLC and adjacent normal tissues. SMAD family member 3 (SMAD3) was considered as a novel target gene by luciferase reporter assay and western blot in NSCLC. The present study demonstrated that miR-32-5p is frequently downregulated in NSCLC tissues. The overexpression of miR-32-5p resulted in the inhibition of migratory and invasive abilities in NSCLC cells. Thus, SMAD3 was identified as a target of miR-32-5p, and its expression was negatively correlated with miR-32-5p expression in clinical NSCLC tissues. Overall, these findings indicate that miR-32-5p serves as a tumor suppressor by targeting SMAD3. Thus, miR-32-5p may be a potential therapeutic target for the treatment of lung adenocarcinoma.

## Introduction

Lung cancer is the leading cause of cancer-associated death, according to the latest epidemiological data 1. According to the diverse pathobiological features, lung cancer can be divided to small cell lung cancer (SCLC) and non-small cell lung cancer (NSCLC), where NSCLC accounts for ~85% of all lung cancer and it more easily metastasizes 1, 2. The 5-year survival rate of patients remains 10%, and most cases of NSCLC are diagnosed at an advanced clinical stage 3,4. NSCLC is further divided into lung adenocarcinoma (LUAD), lung squamous carcinoma (LUSC) and large cell carcinoma (LCC) based on their histological features 5. LUAD is the most common pathological type of lung cancer. Although, for the LUAD, significant improvements in progression-free survival (PFS) have been achieved for patients with epidermal growth factor receptor (EGFR) mutations, anaplastic lymphoma kinase (ALK) translocation or other mutations due to the effectiveness and availability of therapies that target these molecular drivers. Consequently, drug resistance has become the poster child of targeted therapy in oncology 6. Therefore, it is necessary for patients with NSCLC to understand the mechanisms understanding the process of NSCLC and find more putative therapy targets.

MicroRNAs (miRNAs) are a group endogenous non-coding small RNAs, of ~22 nucleotides, which are widely reported in a variety of cancer types 7-10. Several miRNAs, including miR-21, miR-23a, miR-410, miR-135, have shown potential to reverse the PI3K/AKT/mTOR pathway-induced inhibition of tumor growth, progression, and metastasis 5. A previous study demonstrated that miR-302a-5p inhibits the proliferation and invasion in NSCLC by targeting ITGA6 8. miR-337, as a tumor suppresser, has been reported to prevent the migration and invasion of NSCLC cells 11. Ye *et al.* 12,13 reported lower expression of miR-32-5p in cervical cancer tissues and inhibition of cell proliferation and invasion, by targeting HOXB8. Besides, miR-32-5p has been reported to be downregulated in prostate cancer and lower expression of miR-32-5p is associated with cisplatin 14,15. In hepatocellular carcinoma, decreased miR-32-5p expression has been reported in tumor tissues compared with adjacent normal tissues, and the level of miR-32-5p is markedly associated with tumor stage, size and lymph node metastasis. Kaplan-Meier survival analysis revealed a high 5-year survival rate in patients with high expression of miR-32-5p compared with those with lower expression level of miR-32-5p 16.

SMAD family member 3 (SMAD3) is a major component of TGF-β signaling pathway, which leads to the activation and phosphorylation of SMAD3, and promotes the formation of the SMAD2/3/4 complex. The SMAD2/3/4 complex translocates to the nucleus and modulates numerous cancer-associated gene expression such as Snail, ZEB and twist family 17. TGF-β signaling regulates the transcription of >500 genes, which may contain one or more Smad binding elements (SBEs). Furthermore, the activity and stability of SMAD protein are regulated by post-transcription/translation modifications 18. Hu *et al.* 19 reported that miR-145 and miR-203 inhibits the epithelial-mesenchymal transition and invasion in NSCLC, by binding to the 3' untranslated region, decreasing the expression of SMAD3. In the present study, miR-32-5p was reported to be downregulated in NSCLC tissues compared with adjacent tissues and the decreased expression is associated with poor overall survival. Furthermore, overexpression of miR-32-5p prevents migration and invasion in NSCLC; whereas the knockdown of miR-32-5p promotes these processes.

## Materials and methods

### Cell culture

Human lung adenocarcinoma cell lines A549 and H1299 were purchased from the Cell Bank of Chinese Academy of Sciences. The cells were cultured in RPMI-1640 medium (Gibco; Thermo Fisher Scientific, Inc.), supplemented with 1% penicillin and streptomycin, and 10% fetal bovine serum (FBS) (Gibco; Thermo Fisher Scientific, Inc.) at 37 °C in a 5% CO_2_ atmosphere.

### Western blotting

Protein was collected and extracted from A549 and H1299 cells with RIPA lysis buffer and protease inhibitor cocktail and protein phosphatase inhibitor. The samples were then transferred to PVDF membranes, following electrophoresis and the membranes were incubated with rabbit-anti SMAD3 (Cell Signaling Technology, Inc.) or mouse-anti E-cadherin or mouse-anti N-cadherin (both Santa Cruz Biotechnology, Inc.) primary antibodies(SC-8426 for E-cadherin, SC-8424 for N-cadherin) overnight at 4 °C. The following day, the membranes were incubated with indicated secondary antibodies (Santa Cruz Biotechnology, Inc, SC-2005) for 1 h at room temperature. Detection was performed using the electrochemiluminescence kit (Pierce; Thermo Fisher Scientific, Inc.). β-actin (Santa Cruz Biotechnology, Inc.) was used as the internal control.

### Reverse transcription-quantitative PCR (RT-qPCR)

For the detection of miR-32-5p expression, total RNA was extracted from A549 cells using TRIzol, according to the manufacturer's instruction (Thermo Fisher Scientific, Inc.). Synthesis of cDNA with reverse transcriptase was performed with TaqMan miRNA assays (Applied Biosystems; Thermo Fisher Scientific, Inc.) for miRNA and M-MLV First Strand Kit (Thermo Fisher Scientific, Inc.) for mRNA. Real-time PCR analysis was carried out using Green Kit (Takara Biotechnology Co., Ltd.). U6 and GAPDH was used as the internal controls, respectively. The primer sequences were listed in Table 1. The 2^-ΔΔCq^ method was used to determine the relative expression of SMAD3, CDH2, PAI-1, Snail and miR-32-5p expression.

### Dual luciferase reporter assay

The 3' untranslated region (UTR) of SMAD3 containing miR-32-5p binding site and the mutated sequences were synthesized into the psicheck-2 plasmid. Subsequently, A549 cells were seeded into 24-well plate and co-transfected with 50 ng of psicheck-2-SMAD3-3'UTR wild-type or mutant-type vectors and with 20 nM of either miR-159-5p mimic or miR negative control (miR-NC). After 48 h, cells were collected, and the luciferase activities were measured using the Dual-Luciferase Reporter Assay Kit (Promega Corporation) following the direction of the user manual.

### Transwell assay

After transfecting with miR-NC or miR-32-5p, A549 cells were added into the upper chamber of the Transwells for migration assay, and for invasion assay, the upper chamber was pre-coated with Matrigel. The lower chamber contained 800 ul medium with 20% FBS. Subsequently, the cells were collected and fixed with 4% polyformaldehyde for 20 min and then stained with crystal violet. The images of migrated and invaded cells were captured and counted by inverted microscope.

### Generation of a stable cell line overexpressing miR-32-5p

To generate A549 cells, in which miR-32-5p can be stably overexpressed, a 500-bp amplified DNA fragment containing a primary hsa-miR-32-5p transcript was subcloned into a pLVX-IRES-Neo vector by restriction endonuclease XhoI and XbaI for expression through a Lenti-X expression system (Clontech Laboratories, Inc.). Subsequently, the miR-32-5p overexpression vector or empty vector were co-transfected with packaging plasmids into human embryonic kidney (HEK) 293T cells using Lipofectamine 3000 (Thermo Fisher Scientific, Inc.). The empty vector was used as negative control (miR-neg). After 48 h, the packaged lentiviruses were harvested and used to infect A549 cells. The infected cells were cultured in medium for two days, and stable cells were selected by adding 300 μg/ml G418 (Thermo Fisher Scientific, Inc.).

### Construction of flag-tagged SMAD3 expression vectors

The coding sequence of SMAD3 (NM_005902.3) was subcloned into the empty PCDH-flag vector using the restriction enzymes NheI and BamHI (Thermo Fisher Scientific, Inc.). The sequence of plasmid construct was confirmed by direct sequencing before transfection.

### NSCLC tissue samples

A total of 24 NSCLC tissues were obtained after informed consent was obtained from patients in The First Affiliated Hospital of Nanjing Medical University between 2015 and 2018. Pathological diagnostics for patients with NSCLC were assessed according to the Revised International System for Staging Lung Cancer. This study was approved by the Ethics Review Board of Nanjing Medical University.

### *In vivo* experiments of metastasis assays

A total of 10 female BALB/c athymic nude mice (age, 4-6 weeks old; weight, 16-20 g) were purchased from the Experimental Animal Center of Nanjing Medical University and bred under pathogen-free conditions. Mice were maintained in exhaust ventilated closed system cages in a specific pathogen-free environment, with 55±5% humidity, at 23±2 °C, ad libitum access to food and water, and a 14/10 h light/dark cycle. Mice were divided into two groups, termed as miR-32-5p-overexpression group and control group (5 mice per group). miR-32-5p-overexpression and control A549 cells (2.5 × 10^6^ cells/mouse) in 150-ul of PBS were intravenously (i.v.) injected into the lateral tail vein of mice. There were no deaths cases during the experiment. We observed the mice every seven days and monitored the changes of appetite, mental state and inoculation site of animals. Six weeks later, the mice were narcotized by sodium pentobarbital at a dosage of 60 mg/kg and sacrificed by cervical dislocation. Their lung tissues were taken out and fifixated in Bouin's fluid, and macroscopically observable metastatic nodules on surface of each tissue were counted. Lung tissues were histologically analyzed with H&E staining for the presence of tumor cell micrometastases. Animal studies were approved by the Ethics Committee of Nanjing Medical University.

### Database analysis

Oncomine database (https://www.oncomine.org) is a web-based gene chip data-mining platform consisting of microarray databases covering 715 microarray datasets and 86,733 cancer and normal tissue samples. We used Oncomine database to analyze the mRNA expression of SMAD3 in LUAD. Kaplan-Meier plotter (http://kmplot. com/analysis) contains information on 54,675 genes and 10,188 cancer samples, including lung (n = 3452). It can be used to verify the impact of biomarker genes on survival. In the current study, this tool is used to evaluate the prognostic value of different SMAD3 expression. The Gene Expression Omnibus (GEO) (www.ncbi.nlm.nih.gov/) dataset stores curated gene expression DataSets, as well as original Series and Platform records in GEO repository. In this study, we used the database to analyze the expression of SMAD3 in LUAD samples. The starBase (http://starbase.sysu.edu.cn/) is database which can be used to identify the RNA-RNA and protein-RNA interaction networks from 108 CLIP-Seq (PAR-CLIP, HITS-CLIP, iCLIP, CLASH) data sets generated by 37 independent studies. In this study, the database was used to identify the target genes of miR-32-5p and SMAD3 was selected in the genes.

### Statistical analysis

Results are presented as mean ± SD. All statistical analyses were performed using GraphPad Prism 5.02 (GraphPad Software) and SPSS 16.0 software (SPSS, Inc.). The significance among multiple groups was evaluated using one-way ANOVA followed by Newman‑Keuls (SNK) t‑test. Significant differences between 2 groups (parametric) were analyzed using a Student's t-test, and significant differences between 2 groups (non‑parametric) were analyzed by the Mann‑Whitney U test. Pearson's correlation coefficient test was performed to evaluate the correlation between SMAD3 and miR-32-5p. P<0.05 was considered to indicate a statistically significant difference. All experiments were repeated three times independently.

## Results

### miR-32-5p overexpression decreases migration and invasion of LUAD cells

**Figure 1:**
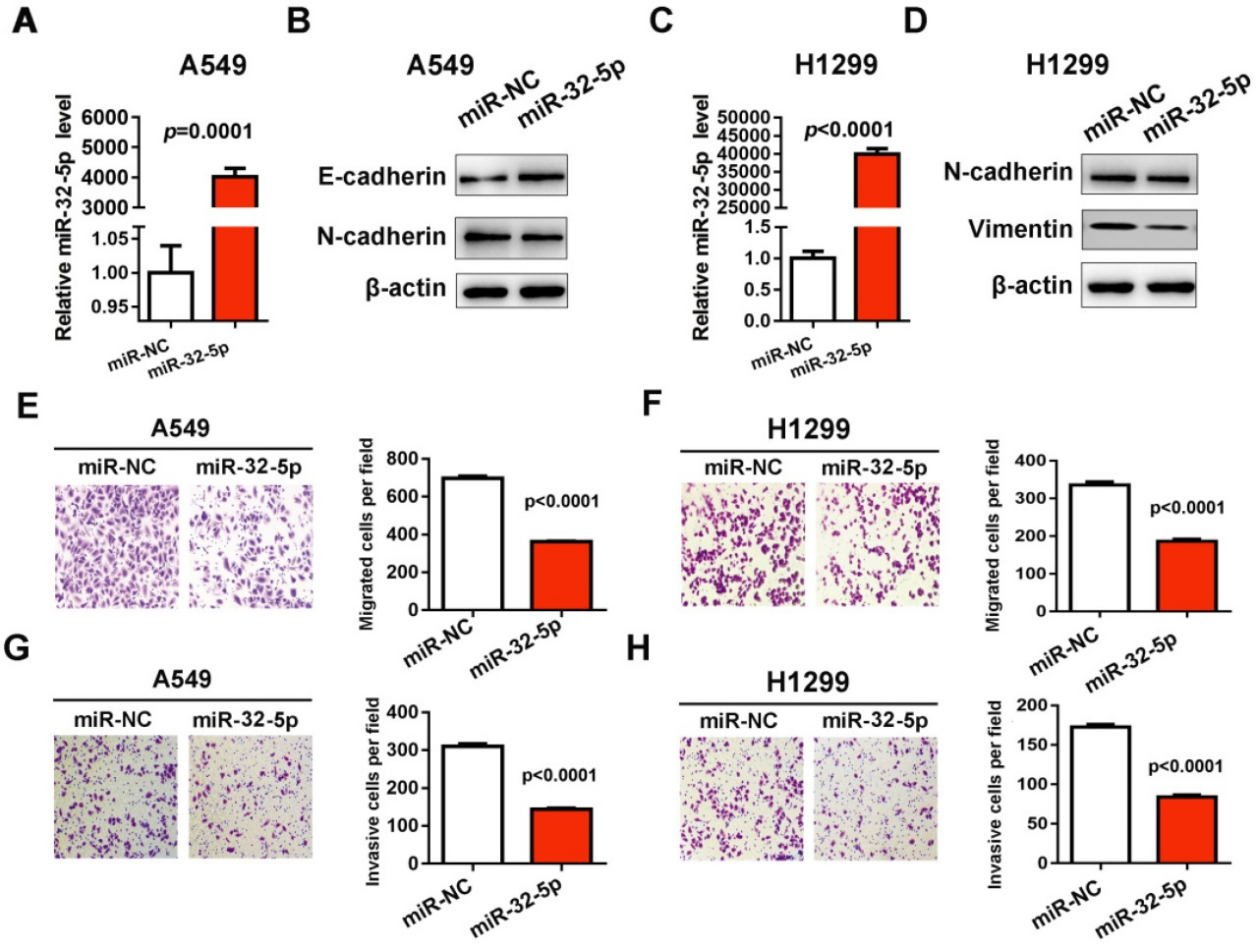
** miR-32-5p overexpression decreases migration and invasion in A549 cells.** (A) The expression level of miR-32-5p in A549 cells transfected with miR-32-5p mimic or miR-NC was detected by RT-qPCR. U6 was used as the internal control. (B) The expression levels of E-cadherin, N-cadherin were analyzed in A549 cells transfected the mimics of miR-32-5p or miR-NC by western blotting. β-actin was used as the internal control. (C) The expression level of miR-32-5p in H1299 cells transfected with miR-32-5p mimic or miR-NC was detected by RT-qPCR. (D) The expression levels of E-cadherin, N-cadherin and vimentin were analyzed in A549 and H1299 cells transfected the mimics of miR-32-5p or miR-NC by western blotting. (E and F) miR-32-5p overexpressed A549 and H1299 cells were allowed to migrate through an 8-µm pore in Transwells. Migrated cells were stained and counted in three microscopic fields (left). And the average cell number was compared between the cells transfected miR-NC and miR-32-5p (right). (G and H) A549 and H1299 cells transfected with miR-NC or miR-32-5p were allowed to invade through the Matrigel-coated membrane in Transwells. Invasive cells were stained, and the average cell number was compared between the two groups. Data are shown as the mean ± SD. ^***^P<0.01. miR, microRNA; RT-qPCR, reverse transcription-quantitative PCR; NC, negative control.

In order to investigate the effect of miR-32-5p on cell migration and invasion in NSCLC, the mimics of miR-32-5p were synthetized and transfected into A549 and H1299 cells. RT-qPCR verified that the mimics of miR-32-5p were successfully transfected into the A549 cells (Fig. 1A and C). Subsequently, the effects of miR-32-5p overexpression on EMT were investigated, by determining the expression levels of EMT markers in A549 and H1299 cells. miR-32-5p overexpression in A549 and H1299 cells resulted in the upregulation of epithelial cells marker E-cadherin and the downregulation of mesenchymal cells markers N-cadherin, vimentin and compared with cells transfected with miR-NC (Fig. 1B and D). Next, we analyzed the effects of miR-32-5p on the migration and invasion of A549 cells. The overexpression of miR-32-5p significantly promoted the inhibition of migration (Fig. 1E and F) and invasion (Fig. 1G and H) in A549 and H1299 cells. These results suggest that miR-32-5p overexpression inhibits EMT and migration in LUAD cells.

### miR-32-5p knockdown promotes migration and invasion in A549 cells

**Figure 2:**
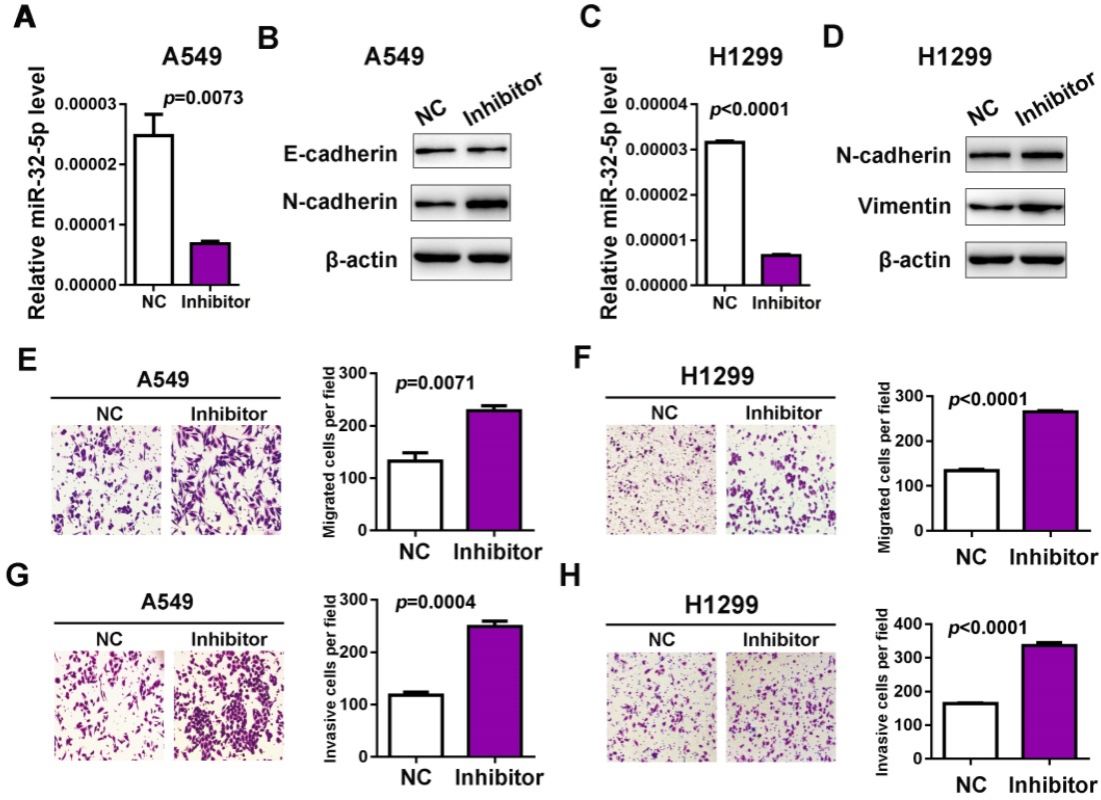
** Knockdown miR-32-5p promotes migration and invasion in A549 cells.** (A and C) The expression level of miR-32-5p in A549 and H1299 cells transfected with miR-32-5p inhibitor or NC was detected by reverse transcription-quantitative PCR. (B and D) The expression levels of E-cadherin, N-cadherin and vimentin were analyzed in A549 and H1299 cells transfected with miR-32-5p inhibitor or NC by western blotting. (E and F) miR-32-5p-silenced A549 and H1299 cells were allowed to migrate through an 8-µm pore in Transwells. Migrated cells were stained and counted in three microscopic fields (left). The average cell number was compared between the cells transfected with miR-NC and miR-32-5p (right). (G and H) A549 and H1299 cells transfected with NC or miR-32-5p inhibitor were allowed to invade through the Matrigel-coated membrane in Transwells. Invasive cells were stained and the average cell number was compared between the two groups. Data are shown as the mean ± SD. ***P<0.01 vs. control. Inhibitor, the inhibitor of miR-32-5p; miR, microRNA; NC, negative control.

Next, the inhibitor of miR-32-5p was transfected into A549 cells and qRT-PCR was used to confirm the knockdown of miR-32-5p (Fig. 2A and C). The effects of the miR-32-5p inhibitor on EMT markers were analyzed. The miR-32-5p inhibitor, resulted in the downregulation of epithelial cell marker E-cadherin and the upregulation of mesenchymal cell marker N-cadherin, vimentin compared with miR-NC-transfected A549 and H1299 cells (Fig. 2B and D). Subsequently, the migration and invasion capabilities were detected in cells transfected with miR-32-5p inhibitor. The knockdown of miR-32-5p markedly promoted the migration and invasion in A549 and H1299 cell lines compared with cells transfected with inhibitor-NC (Fig. 2E-H). These results demonstrate that miR-32-5p suppression promotes EMT, migration and invasion abilities in LUAD cells.

### Overexpression of miR-32-5p suppress LUAD metastasis *in vivo*

**Figure 3:**
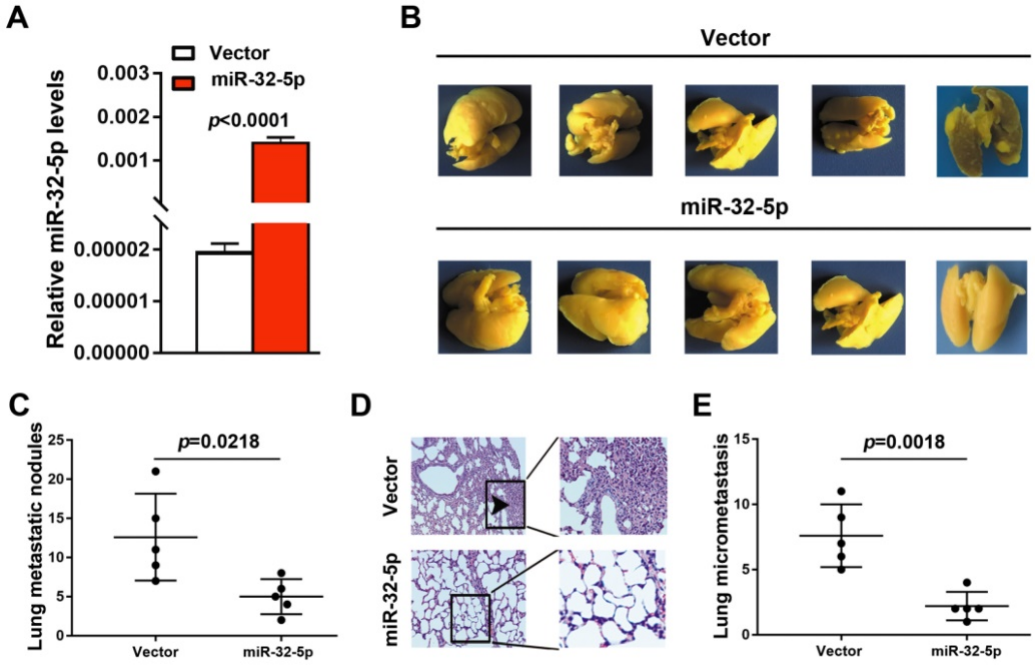
** miR-32-5p suppresses non-small cell lung cancer metastasis *in vivo.*** (A) Reverse transcription-quantitative PCR analysis of miR-32-5p expression in A549 stable cells (miR-32-5p) and negative control A549 cells (vector). (B) Representative images showing metastatic nodules generated in lung tissues resected from mice which were injected with empty vector- and miR-32-5p-A549 cells. The resected lung tissues were fix and stained in Bolin's fluid for 24 h and the manifested metastatic nodules were counted manually. Black arrowheads indicate metastatic nodules. (C) Comparison of the number of metastatic lung nodules between empty vector group and miR-32-5p-overexpressed group. Unpaired t-test was used, and results are presented as mean ± SD. ^*^P<0.05 vs. control. (D) Representative images of hematoxylin and eosin staining in paraffin-embedded sections of Bolin's fluid-fixed tissues mentioned in (B) and micrometastases sites were accounted. ^***^P<0.001. miR, microRNA; NC, negative control.

To further detect the effect of miR-32-5p on LUAD metastasis *in vivo*, A549 cells stably overexpressing miR-32-5p was established (Fig. 3A). Subsequently, the miR-32-5p-overexpressed stable A549 cells (miR-32-5p) or empty-vector-stable A549 cells (vector) were injected i.v. into the tail vein of BALB/c nude mice. Eight weeks after injection, lung tissues were surgically obtained, and then fixed and stained using Bolin's fluid, which allows the metastatic nodules to be more apparent and visible. As shown in Fig. 3B and C, more pulmonary metastasis nodules on the lung surface were observed in mice injected with empty-vector-transfected cells compared with those injected with miR-32-5p-overexpressed cells. Then, pulmonary micrometastases were histologically detected by hematoxylin-eosin (H&E) staining in all mice. Results showed that more pulmonary micrometastases were detected in mice injected with empty-vector cells compared with those injected with miR-32-5p-overexpressed cells (Fig. 3D and E). Collectively, the *in vivo* experiment of metastasis shows that overexpression of miR-32-5p inhibits pulmonary metastasis of LUAD cells, which is consistent with the present *in vitro* findings.

### miR-32-5p directly targets SMAD3 in LUAD

**Figure 4:**
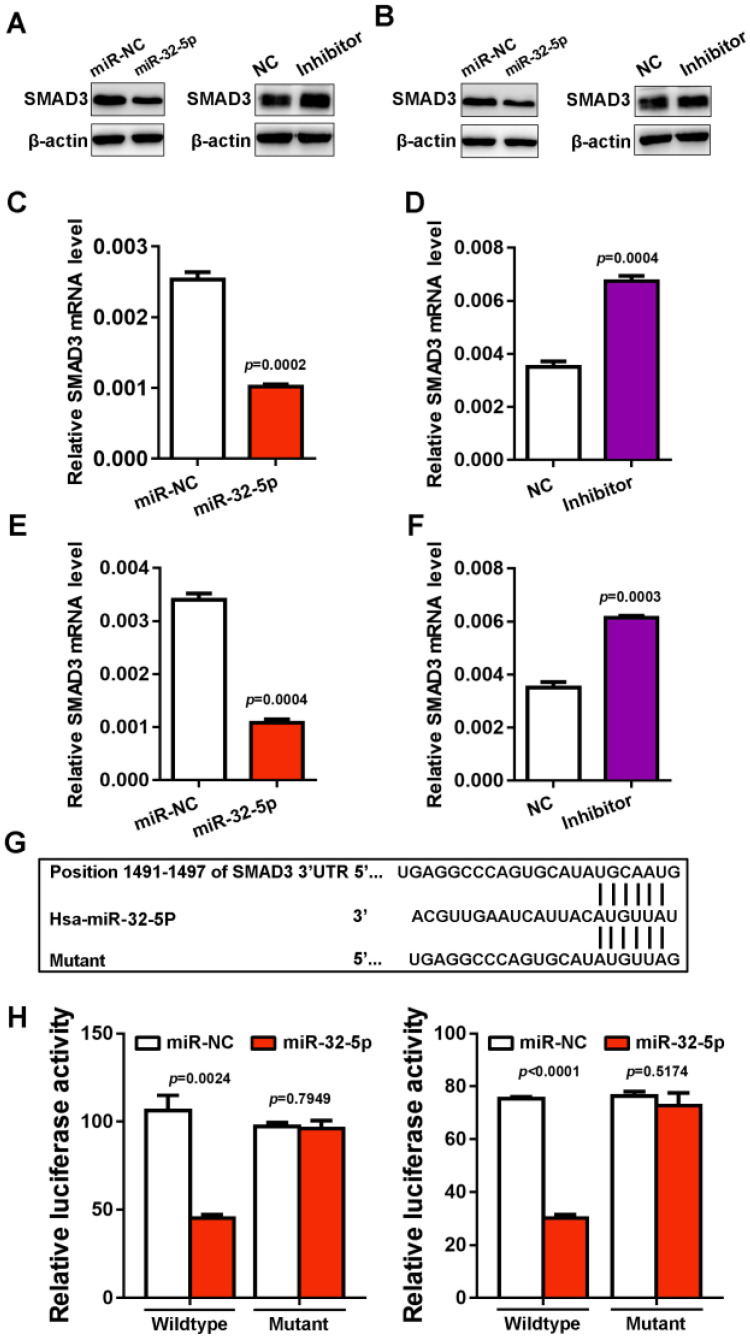
** SMAD3 is a direct target gene of miR-32-5p.** (A and B) The expression level of SMAD3 was detected in A549 and H1299 cells transfected with mimics or the inhibitor of miR-32-5p or NC by western blotting. (C-F) The mRNA level was quantified by reverse transcription-quantitative PCR in A549 cells transfected with mimics or the inhibitor of miR-32-5p or NC. (E) The luciferase activity of cells was examined via the dual-luciferase reporter assay in A549 and H1299 cells after co-transfecting the construct containing the wild-type- or mutant-SMAD3 reporter gene with miR-32-5p or miR-NC. Data are shown as the mean ±SD. *P<0.05; ***P<0.001 vs. control. miR, microRNA; NC, negative control; SMAD3, SMAD family 3.

To investigate the mechanism by which miR-32-5p suppresses EMT, migration and invasion, the starBase miRNA target prediction program was used, where SMAD3 was found as a putative target of miR-32-5p. To verify the prediction, miR-32-5p mimic was transfected into A549 and H1299 cells, and the expression of SMAD3 was analyzed. The overexpression of miR-32-5p mimic dramatically inhibits the protein level of SMAD3 in A549 and H1299 cells. In contrast, the miR-32-5p inhibitor promoted the expression of SMAD3 in both A549 and H1299 cells (Fig. 4A and D). As well as the level of mRNA, the knockdown of miR-32-5p substantially increased the expression of SMAD3 in A549 and H1299 cells (Fig. 4E and F). Next, the psiCHECK-2 luciferase vectors, which contain wild-type or the mutant of SMAD3 3'UTR (miR-32-5p binding site) (Fig. 4G) were constructed. Then the vectors were co-transfected into A549 and H1299 cells with miR-32-5p mimics. Results show that miR-32-5p mimics significantly downregulated the activity of the wild-type reporter compared with cells transfected with miR-NC (Fig. 4F and I). At the same time, miR-32-5p mimics had no effect on the luciferase activity of the mutant type, in contrast to the miR-NC group (Fig. 4F and I). Taken together, miR-32-5p binds to the 3' UTR region of SMAD3 and inhibits its expression in LUAD cells.

### miR-32-5p regulates SMAD3-mediates genes transcription in LUAD

**Figure 5:**
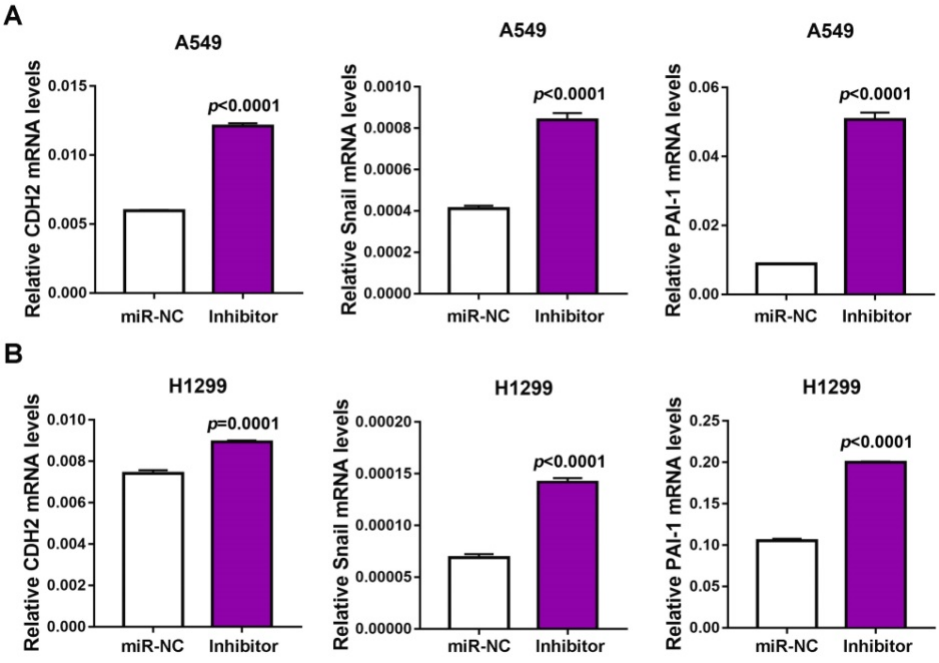
** miR-32-5p regulates SMAD3-mediates genes transcription in NSCLC.** (A and B) miR-NC and inhibitor of miR-32-5p was transfected into A549 and H1299 cells, after 48 h, mRNA was extracted and reverse transcription-quantitative PCR was used to quantify the expression of CDH2, Snail and PAI-1. Data are shown as the mean ± SD. ***P<0.01. miR, microRNA; NC, negative control; Inhibitor; inhibitor of miR-32-5p; SMAD3, SMAD family 3.

SMAD3 is widely reported as a transcript factor in TGF-β signaling pathway, which modulates the expression of cadherin 2 (CDH2), Snail and plasminogen activator inhibitor-1 (PAI-1). To verify whether miR-32-5p affects SMAD3-mediated EMT and metastasis-associated gene transcription, the expression of CDH2, Snail and PAI-1 was quantified, following miR-32-5p downregulation in A549 cells. The expression of CDH2, Snail and PAI-1 were significantly upregulated in miR-32-5p-inhibitor-transfected A549 cells compared with the miR-NC group (Fig. 5A); this was also observed in H1299 cells (Fig. 5B). These results demonstrate that miR-32-5p regulated SMAD3-mediated EMT and metastasis-associated genes transcription in A549 and H1299 cells.

### Overexpression of SMAD3 promotes migration and invasion in LUAD cells

**Figure 6:**
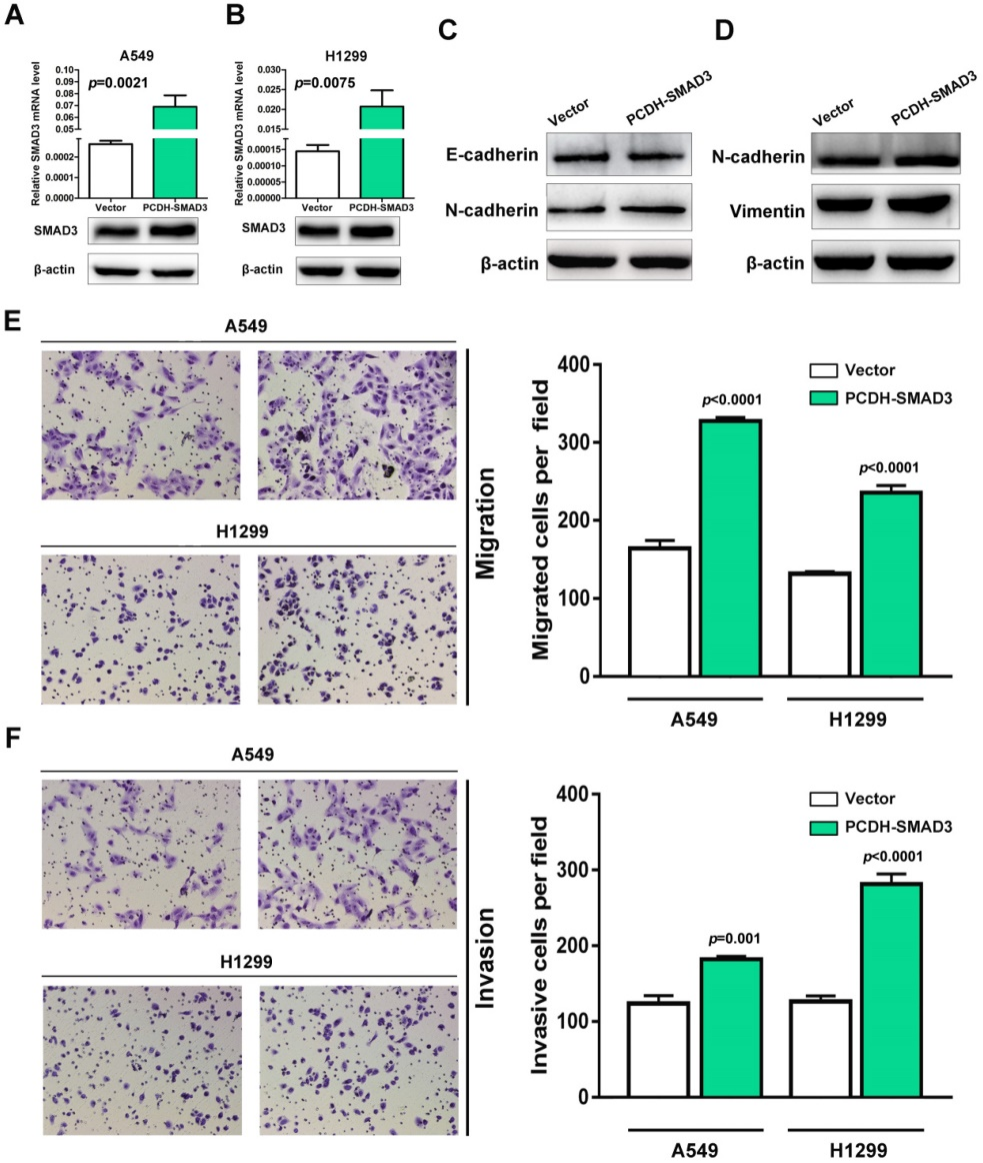
** Overexpression of SMAD3 promotes migration and invasion in NSCLC cells.** (A and B) Empty and PCDH-SMAD3 vectors were transfected into A549 and H1299 cells. The expression of SMAD3 was measured at both mRNA and protein levels. (C and D) The expression levels of E-cadherin, N-cadherin and vimentin were analyzed in A549 and H1299 cells that were transfected with the empty or PCDH-SMAD3 vectors by western blotting. (E and F) A549 and H1299 cells transfected with empty or PCDH-SMAD3 vectors were allowed to invade through the Matrigel-coated membrane in Transwells. Migrated and invaded cells were stained, and the average cell number is compared between the two groups. Data are shown as the mean ± SD. ***P<0.01. SMAD3, SMAD family 3; NSCLC, non-small cell lung cancer.

SMAD3, as a critical mediator of TGF-β signaling pathway, has been reported as a promoter of EMT and migration in multiple cancer types. To test whether SMAD3 promotes the procession of EMT and metastasis in NSCLC. The expression of SMAD3 was upregulated in A549 and H1299 cells. As shown in Fig. 6A and B, SMAD3 was successfully upregulated at both mRNA and protein levels in A549 and H1299 cells. To verify the effects of SMAD3 overexpression on EMT, the expression of EMT-associated markers was determined in A549 and H1299 cells. Western blotting showed that epithelial cell marker E-cadherin was downregulated and mesenchymal cell markers N-cadherin and vimentin were upregulated significantly in SMAD3-overexpressed A549 and H1299 cells (Fig. 6C and D). Next, transwell assay was performed to explore the effect of SMAD3 on the abilities of migration and invasion in A549 and H1299 cells. As shown in figure 6E and F, the overexpression of SMAD3 promotes the abilities of migration and invasion in A549 and H1299 cells. Taken together, these results demonstrate that SMAD3 promotes EMT and metastasis in LUAD cells.

### miR-32-5p is downregulated in NSCLC and is correlated with SMAD3

**Figure 7:**
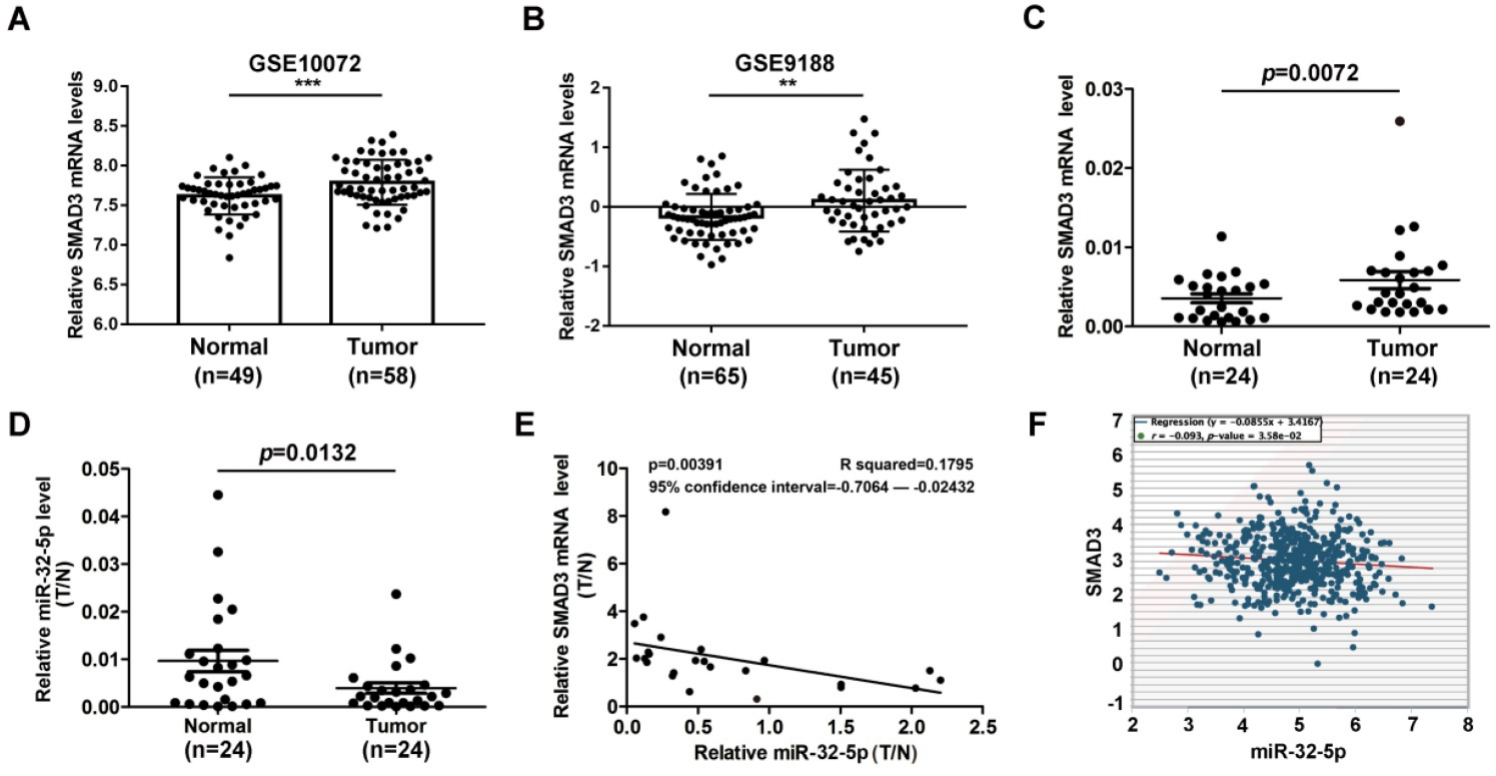
** miR-32-5p is downregulated in NSCLC and is associated with survival.** (A and B) The data from the GEO database revealed that SMAD3 in upregulated in NSCLC tissues compared with normal tissues. (C and D) The expression levels of SMAD3 and miR-32-5p in NSCLC tissues and normal tissues was detected by reverse transcription-quantitative PCR. (E) The correlation between miR-32-5p and SMAD3 expression was analyzed by Pearson's correlation analysis in 24-paired NSCLC tissues. (F) The correlation between miR-32-5p and SMAD3 expression in NSCLC tissues from Starbase database. Data are shown as the mean ±SD. *P<0.05; **P<0.01; ***P<0.001 vs. control. NSCLC, non-small cell lung cancer; SMAD3, SMAD family 3; miR, microRNA; NC, negative control.

miR-32-5p has been well known as an inhibitor of tumor invasion and metastasis in multiple cancer types. Meanwhile, SMAD3 has been well known as a metastasis-inducer in a series of cancer types. There is little evidence that reveals the correlation between miR-32-5p and SMAD3 in patients with NSCLC. To explore the correlation between miR-32-5p and SMAD3 in patients with NSCLC, the expression level of miR-32-5p was analyzed in the Gene Expression Omnibus database. SMAD3 was dramatically upregulated in LUAD tissues compared with normal tissues (Fig. 7A and B). Subsequently, the expression of miR-32-5p and SMAD3 was quantified in 24 paired-NSCLC tissues. As shown in Fig. 7C and D, SMAD3 was upregulated in tumor tissues compared with the adjacent normal lung tissues. In contrast, miR-32-5p was downregulated in tumor tissues compared with the adjacent normal lung tissues. Furthermore, Pearson's correlation analysis demonstrated a significant negative correlation between the expression of miR-32-5p and SMAD3 mRNA expression in NSCLC tissues (Fig. 7E). Furthermore, data from starBase (http://starbase.sysu.edu.cn) also showed significant negative correlation between the expression of SMAD3 and miR-32-5p mRNA (Fig. 7F). Overall, the results of the present study demonstrate the crucial role of miR-32-5p in patients with LUAD.

## Discussion

miRNAs have been considered as critical components in the process of cancer growth and metastasis 20,21. miR-32-5p was widely described as a tumor suppresser of various cancer including thyroid cancer, colorectal cancer and pancreatic cancer 22-27. Recently Wang *et al.* demonstrated that miR-195-5p was a biomarker in clean cell renal carcinoma and inhibits the proliferation in clean cell renal carcinoma by preventing TR4 25,28. However, whether miR-32-5p affects the migration and metastasis of NSCLC cells is still unknown. In the present study, it was revealed that miR-32-5p act as an EMT inhibitor by targeting SMAD3 in NSCLC cells. Meanwhile, miR-32-5p significantly suppressed the migration of A549 cells *in vivo*. Taken together, this study first revealed that miR-32-5p inhibits EMT and metastasis by targeting SMAD3 and modulates SMAD3-mediated oncogene expression in NSCLC.

In fact, many miRNAs have been demonstrated to be involved in the process of NSCLC development. For instance, miR-363-3p has been revealed as an inducer of invasion and metastasis, through modulating the expression of NEDD9 and SOX4 in NSCLC 29. Besides, Gan *et al.* 30 reported that miR-325-3p facilitated the proliferation of NSCLC cells by regulating KIF2C level. miR-32-5p was also reported to be downregulated in several types of cancer, including NSCLC, suggesting its inhibitory role in NSCLC metastasis 31-33. In the present study, it was found that knockdown of the endogenesis miR-32-5p significantly prevented the migration and invasion of NSCLC. By contrary, overexpression of miR-32-5p dramatically promotes the migratory and invasive abilities in NSCLC cells. Overall, the present study demonstrated a critical role for miR-32-5p in the invasion and metastasis for the first time.

SMAD3 is a central component of TGF-β signaling pathway, which has been reported to exerts tumor-suppressive actions that include inhibition of cellular proliferation and immortalization, and in also promotes apoptosis in normal cells and early carcinomas but promotes EMT and procession in various cancer types 34-36. Recently, miR-5590-3p has been reported as a negative regulator of TGF-β/SMAD3 signaling pathway, by inhibiting SMAD3 in breast cancer 37. Also, Zhang *et al.* 38 demonstrated that SMAD3 is a downstream target of miR-16-5p in chordoma. In NSCLC, studies have shown that miR-145 and miR-203 prevent EMT and invasion by targeting SMAD3 19. The present study analyzed the data from TCGA in Ocomine database and the results revealed that SMAD3 was highly expressed in tumor tissues compared with normal tissues (data not shown). Furthermore, data from Kaplan-Meier plotter database showed that overexpressed SMAD3 was correlation with poor survival in patients with NSCLC (data not shown). Taken together, these findings support the notion that SMAD3 has an important role in tumor metastasis and survival.

## Figures and Tables

**Table 1:** Primers for RT-qPCR

Name	Sequence, 5'-3'
miR-32-5p	F:CGGTATTGCACATTACTAAGTTGCA
	R:TCCAGTGCGTGTCGTGGA.
U6	F:CGAGCACAGAATCGCTTCA
	R:CTCGCTTCGGCAGCACATAT.
SMAD3	F:CCATCTCCTACTACGAGCTGAA
	R:CACTGCTGCATTCCTGTTGAC.
Snail	F: TCGGAAGCCTAACTACAGCGA
	R: AGATGAGCATTGGCAGCGAG.
PAI-1	F: ACCGCAACGTGGTTTTCTCA
	R:TTGAATCCCATAGCTGCTTGAAT.
CDH2	F: TCAGGCGTCTGTAGAGGCTT
	R:ATGCACATCCTTCGATAAGACTG.

F: forward R: reverse.

## References

[1] Huang W, Yan Y, Liu Y, Lin M, Ma J, Zhang W (2020). Exosomes with low miR-34c-3p expression promote invasion and migration of non-small cell lung cancer by upregulating integrin alpha2beta1. Signal Transduct Target Ther.

[2] Bray F, Ferlay J, Soerjomataram I, Siegel RL, Torre LA, Jemal A (2018). Global cancer statistics 2018: GLOBOCAN estimates of incidence and mortality worldwide for 36 cancers in 185 countries. CA: a cancer journal for clinicians.

[3] Chen Z, Fillmore CM, Hammerman PS, Kim CF, Wong KK (2014). Non-small-cell lung cancers: a heterogeneous set of diseases. Nat Rev Cancer.

[4] Wang X, Adjei AA (2015). Lung cancer and metastasis: new opportunities and challenges. Cancer Metastasis Rev.

[5] Yuan M, Huang LL, Chen JH, Wu J, Xu Q (2019). The emerging treatment landscape of targeted therapy in non-small-cell lung cancer. Signal Transduct Target Ther.

[6] Vyse S, Huang PH (2019). Targeting EGFR exon 20 insertion mutations in non-small cell lung cancer. Signal Transduct Target Ther.

[7] Yan H, Ren S, Lin Q, Yu Y, Chen C, Hua X (2019). Inhibition of UBE2N-dependent CDK6 protein degradation by miR-934 promotes human bladder cancer cell growth. FASEB J.

[8] Cai J, Chen Z, Wang J, Wang J, Chen X, Liang L (2019). circHECTD1 facilitates glutaminolysis to promote gastric cancer progression by targeting miR-1256 and activating beta-catenin/c-Myc signaling. Cell Death Dis.

[9] Zhang W, Mao S, Shi D, Zhang J, Zhang Z, Guo Y (2019). MicroRNA-153 Decreases Tryptophan Catabolism and Inhibits Angiogenesis in Bladder Cancer by Targeting Indoleamine 2,3-Dioxygenase 1. Front Oncol.

[10] Chen W, Zhuang X, Qi R, Qiao T (2019). MiR-302a-5p suppresses cell proliferation and invasion in non-small cell lung carcinoma by targeting ITGA6. Am J Transl Res.

[11] Zhang J, Gong WH, Li Y, Zhang HY, Zhang CX (2019). Hsa-miR-337 inhibits non-small cell lung cancer cell invasion and migration by targeting TCF7. Eur Rev Med Pharmacol Sci.

[12] Zhao DL, Wu QL (2019). Effect of inhibition to Yes-related proteins-mediated Wnt/beta-catenin signaling pathway through miR-195-5p on apoptosis of gastric cancer cells. Eur Rev Med Pharmacol Sci.

[13] Liu YJ, Zhou HG, Chen LH, Qu DC, Wang CJ, Xia ZY (2019). MiR-32-5p regulates the proliferation and metastasis of cervical cancer cells by targeting HOXB8. Eur Rev Med Pharmacol Sci.

[14] Lin X, Wang S, Sun M, Zhang C, Wei C, Yang C (2019). miR-195-5p/NOTCH2-mediated EMT modulates IL-4 secretion in colorectal cancer to affect M2-like TAM polarization. J Hematol Oncol.

[15] Zhang L, Li X, Chao Y, He R, Liu J, Yuan Y (2018). KLF4, a miR-32-5p targeted gene, promotes cisplatin-induced apoptosis by upregulating BIK expression in prostate cancer. Cell communication and signaling: CCS.

[16] Fu X, Liu M, Qu S, Ma J, Zhang Y, Shi T (2018). Exosomal microRNA-32-5p induces multidrug resistance in hepatocellular carcinoma via the PI3K/Akt pathway. J Exp Clin Cancer Res.

[17] Chen Y, Di C, Zhang X, Wang J, Wang F, Yan JF (2020). Transforming growth factor beta signaling pathway: A promising therapeutic target for cancer. J Cell Physiol.

[18] Tecalco-Cruz AC, Rios-Lopez DG, Vazquez-Victorio G, Rosales-Alvarez RE, Macias-Silva M (2018). Transcriptional cofactors Ski and SnoN are major regulators of the TGF-beta/Smad signaling pathway in health and disease. Signal Transduct Target Ther.

[19] Hu H, Xu Z, Li C, Xu C, Lei Z, Zhang HT (2016). MiR-145 and miR-203 represses TGF-beta-induced epithelial-mesenchymal transition and invasion by inhibiting SMAD3 in non-small cell lung cancer cells. Lung Cancer.

[20] Wilk G, Braun R (2018). Integrative analysis reveals disrupted pathways regulated by microRNAs in cancer. Nucleic Acids Res.

[21] Chen Y, Min L, Ren C, Xu X, Yang J, Sun X (2017). miRNA-148a serves as a prognostic factor and suppresses migration and invasion through Wnt1 in non-small cell lung cancer. PloS one.

[22] Du P, Liu F, Liu Y, Shao M, Li X, Qin G (2020). Linc00210 enhances the malignancy of thyroid cancer cells by modulating miR-195-5p/IGF1R/Akt axis. J Cell Physiol.

[23] Forouzan Jahromi Z, Javeri A, Fakhr Taha M (2019). Tumor suppressive effects of the pleiotropically acting miR-195 in colorectal cancer cells. EXCLI J.

[24] Zhou WY, Zhang MM, Liu C, Kang Y, Wang JO, Yang XH (2019). Long noncoding RNA LINC00473 drives the progression of pancreatic cancer via upregulating programmed death-ligand 1 by sponging microRNA-195-5p. J Cell Physiol.

[25] Wang M, Sun Y, Xu J, Lu J, Wang K, Yang DR (2018). Preclinical studies using miR-32-5p to suppress clear cell renal cell carcinoma metastasis via altering the miR-32-5p/TR4/HGF/Met signaling. International journal of cancer.

[26] Schneider A, Victoria B, Lopez YN, Suchorska W, Barczak W, Sobecka A (2018). Tissue and serum microRNA profile of oral squamous cell carcinoma patients. Sci Rep.

[27] Gao ZQ, Wang JF, Chen DH, Ma XS, Wu Y, Tang Z (2017). Long non-coding RNA GAS5 suppresses pancreatic cancer metastasis through modulating miR-32-5p/PTEN axis. Cell Biosci.

[28] Zheng J, Xu T, Chen F, Zhang Y (2019). MiRNA-195-5p Functions as a Tumor Suppressor and a Predictive of Poor Prognosis in Non-small Cell Lung Cancer by Directly Targeting CIAPIN1. Pathol Oncol Res.

[29] Chang J, Gao F, Chu H, Lou L, Wang H, Chen Y (2020). miR-363-3p inhibits migration, invasion, and epithelial-mesenchymal transition by targeting NEDD9 and SOX4 in non-small-cell lung cancer. J Cell Physiol.

[30] Gan H, Lin L, Hu N, Yang Y, Gao Y, Pei Y (2019). KIF2C exerts an oncogenic role in nonsmall cell lung cancer and is negatively regulated by miR-325-3p. Cell Biochem Funct.

[31] Chai L, Kang XJ, Sun ZZ, Zeng MF, Yu SR, Ding Y (2018). MiR-497-5p, miR-195-5p and miR-455-3p function as tumor suppressors by targeting hTERT in melanoma A375 cells. Cancer Manag Res.

[32] Luo Q, Zhang Z, Dai Z, Basnet S, Li S, Xu B (2016). Tumor-suppressive microRNA-195-5p regulates cell growth and inhibits cell cycle by targeting cyclin dependent kinase 8 in colon cancer. Am J Transl Res.

[33] Xu H, Hu YW, Zhao JY, Hu XM, Li SF, Wang YC (2015). MicroRNA-195-5p acts as an anti-oncogene by targeting PHF19 in hepatocellular carcinoma. Oncology reports.

[34] Fu Q, Zhang Q, Lou Y, Yang J, Nie G, Chen Q (2018). Primary tumor-derived exosomes facilitate metastasis by regulating adhesion of circulating tumor cells via SMAD3 in liver cancer. Oncogene.

[35] Cheng X, Xu S, Pan J, Zheng J, Wang X, Yu H (2019). MKL1 overexpression predicts poor prognosis in patients with papillary thyroid cancer and promotes nodal metastasis. J Cell Sci.

[36] Jiang L, Wang R, Fang L, Ge X, Chen L, Zhou M (2019). HCP5 is a SMAD3-responsive long non-coding RNA that promotes lung adenocarcinoma metastasis via miR-203/SNAI axis. Theranostics.

[37] Abedini Bakhshmand E, Soltani BM (2019). Regulatory effect of hsa-miR-5590-3P on TGFbeta signaling through targeting of TGFbeta-R1, TGFbeta-R2, SMAD3 and SMAD4 transcripts. Biol Chem.

[38] Zhang H, Yang K, Ren T, Huang Y, Tang X, Guo W (2018). miR-16-5p inhibits chordoma cell proliferation, invasion and metastasis by targeting Smad3. Cell Death Dis.

